# Histological types of brain tumors diagnosed at the Kenyatta National Hospital between 2016 and 2019: a retrospective study

**DOI:** 10.1007/s12672-024-00893-6

**Published:** 2024-02-18

**Authors:** Samwel Rema Gesaka, Parmenas Minda Okemwa, Philip Maseghe Mwachaka

**Affiliations:** 1 Department of Human Pathology, University of Nairobi, Nairobi, Kenya; 2 Department of Human Anatomy and Medical Physiology, University of Nairobi, Nairobi, Kenya; 3 Neurosurgery Division, Kenyatta National Hospital, Nairobi, Kenya

**Keywords:** Brain tumors, Gliomas, Medulloblastomas, Meningiomas, Kenya

## Abstract

**Purpose:**

To determine the histological types of brain tumors diagnosed at the Kenyatta National Hospital, Nairobi, Kenya.

**Methods:**

This retrospective study retrieved patient-archived records at the Kenyatta National Hospital for the period 2016–2019. The histological types of brain tumors were assessed according to age, sex, and the WHO classification for CNS tumors using the GNU PSPP version 1.6.2-g78a33 software. Results were presented in tables and figures.

**Results:**

During the study period, brain tumors appeared to increase gradually; however, there was a decline in 2018. During the study period, 345 brain tumor records were retrieved. Data on age were missing 33 records; hence, 312 records were included for age analyses. The mean age for the pediatrics and adults was 9 (± 5 SD) and 45 (± 14 SD) years, respectively. 88 (28.2%) and 224 (71.8%) tumors were diagnosed among pediatrics and adults, respectively. Most tumors, 60 (19.2%) were reported in patients aged ≤ 10 years, followed by 55 (17.6%), 48 (15.4%), and 47 (15.1%) in patients aged 31–40, 51–60, and 41–50, years, respectively. In both pediatrics and adults, most tumors were diagnosed in females aged ≤ 10 years and 31–40 years, respectively. Overall, two peaks were observed in patients aged 5–15 years and 40–45 years. Gliomas, 43 (48.9%) and medulloblastomas, 21 (23.9%) were the most common tumors in pediatrics, whereas meningiomas, 107 (47.8%) and gliomas, 70 (31.3%) were the most common tumors in adults. Most pediatric and adult tumors were benign with 50 (56.8%) and 157 (70.1%) cases, respectively. Low-grade gliomas and medulloblastomas were the commonest benign and malignant tumors among pediatrics, with 31 (62%) and 21 (55.3%) cases, respectively. Conversely, meningiomas and high-grade gliomas were the most common benign and malignant tumors in adults, with 106 (67.5%) and 44 (65.7%) cases, respectively.

**Conclusion:**

This study highlights the existing burden of brain tumors in Kenya and data from KNH may be representative of the national burden of BTs. This study lays a foundation for subsequent clinical and epidemiological studies and emphasizes the need to adopt existing reporting standards to help realize a complete picture of the burden of brain tumors in Kenya.

## Introduction

Globally, in 2020, approximately 19.3 million new cancers and 10 million deaths from cancer were reported. Of these, brain tumors (BTs) constituted approximately 308,102 (1.6%) new cases and 251,329 (2.5%) deaths [1]. The prevalence of BTs varies according to sex, age at diagnosis, geographical location, race, histological type, and genetic and environmental risk factors and may exhibit temporal incidence trends [2]. Across all World Health Organization (WHO) regions, the annual number of new cases based on pathology was 654,577 (68%) benign and 302,715 (32%) malignant BTs in 2019 [3]. The WHO African region reported 82,481 (8%) new annual cases of both benign and malignant BTs [3]. According to the GLOBOCAN report (2020), the total new cancer cases and deaths in Kenya were 42,166 and 27,092, respectively [4]. Kenya reported 686 (1.6%) benign and malignant BTs and 560 (2.1%) deaths of all new cancer cases and deaths, respectively [4]. Low reporting may explain the low incidence, prevalence, and mortality rates in some Asian and sub-Saharan African countries, which often lack registries, or the existing registries do not include BTs [2, 5].

Brain tumors exhibit a bimodal age distribution, with peaks in incidence, both in childhood ≤ 5 years and adulthood between 45 and 70 years [2, 6]. Notably, in children under 15 years, primary BTs are the most diagnosed solid tumors [7], whereas metastatic BTs are the eight most frequently isolated malignancy in adults > 40 years [8]. Pilocytic astrocytomas and embryonal tumors are common in children, whereas meningiomas, malignant gliomas, and pituitary adenomas are mainly diagnosed in adults [9, 10]. Meningiomas are the most common benign BTs and are more frequently diagnosed in women. Gliomas are the most common malignant BTs and are more frequently diagnosed in men [11, 12]. Malignant BTs are largely due to brain metastases and can be up to tenfold more common than primary tumors [13]. The increased prevalence of brain metastases is attributed to improved cancer care, and thus, cancer patients live longer with an estimated 20–40% likelihood of developing BTs [14]. Primary cancers of the lung, breast, melanoma, and kidney are most likely to metastasize to the brain. However, any primary cancer can generally metastasize to the brain [15, 16]. Primary malignant BTs also contribute significantly to malignant BTs [15]. In low- and middle-income countries, including Kenya, there are inadequate and noncomprehensive data on the burden of BTs, as evidenced by the limited existence of cancer registries, especially for BTs [2, 5, 17, 18]. A few studies based on cancer registries and hospital records have reported very few BTs in Kenya [19, 20]. However, these studies fail to reveal the burden of BTs in Kenya.

Imaging studies, primarily magnetic resonance imaging and computed tomography, are used to locate BTs [21]. The definitive diagnosis is established by histological examination and further characterization by immunohistochemistry and molecular studies where available [22]. Historically, the diagnosis and classification of BTs have been based solely on the histological presentation of the tumor using light microscopy. The WHO classification of tumors of the central nervous system is used to grade tumors into grade 1, 2, 3, and 4 [22]. The integration of molecular techniques in diagnosing BTs has enabled the molecular classification of BTs and potential improvement in diagnostic accuracy, patient management, and objective monitoring of treatment [22]. The current WHO classification integrates molecular genetics and molecular profiling of BTs with conventional techniques, such as electron microscopy, histology, and immunohistochemistry [23]. However, where molecular resources are unavailable, histological diagnosis is sufficient to diagnose BTs [22, 23]. Therefore, this study aimed to determine the histological types of brain tumors diagnosed in a national referral hospital in Nairobi, Kenya.

## Methods

### Study design and setting

This was a descriptive retrospective study that was conducted at the Kenyatta National Hospital (KNH) in Nairobi, Kenya. KNH is the largest referral, teaching, and research hospital in Kenya, with an 1800-bed capacity. Moreover, it is in the nation’s capital and remains the main center for neurosurgical and neurooncological care in Kenya. As a result, most patients with BTs continue to present at the hospital.

### Source of data and data collection

The data in this study were obtained from brain tumor patient records at KNH. Complete records of patients with BTs in all age groups were included. Data variables included age, sex, histological diagnosis, and the WHO grade of BTs. Data were collected using Microsoft Excel 2019 (Microsoft Corp., Redmond, WA, USA). Patient records were retrieved with the assistance of a health records officer.

### Data analysis

Data entry and cleaning were performed using Microsoft Excel 2019 (Microsoft Corp., Redmond, WA, USA). Statistical analysis of the BTs was performed according to patient age, sex, and histological type, and WHO grade. GNU PSPP version 1.6.2-g78a33 software was used to analyze the data and produce tables and graphs.

### Ethical consideration

Ethical approval for this study was obtained from the Kenyatta National Hospital and the University of Nairobi (KNH/UoN) Ethics and Research Committee (registration number UP506/09/2020). All methods in this study adhered to the relevant guidelines and regulations. Permission to access patient records was obtained from the head of Laboratory Medicine Department and the Laboratory Manager Histopathology at KNH. The need for informed consent was waived by the KNH/UoN Nairobi Ethics and Research Committee as this was a retrospective study.

## Results

### Trends in brain tumors diagnosed between 2016 and 2019

**Fig. 1:**
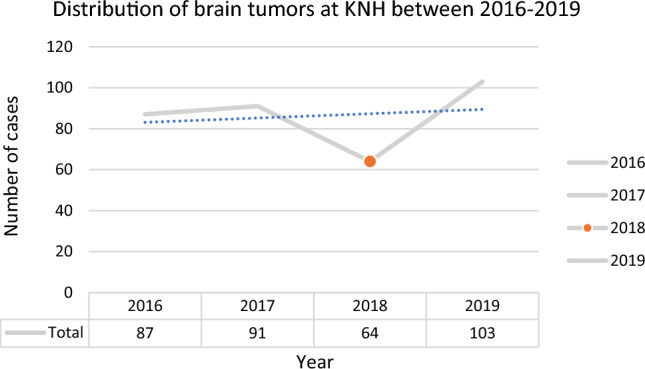
Figure showing trends in brain tumors between 2016 and 2019

87, 91, 64, and 103 brain tumor cases were reported in 2016, 2017, 2018, and 2019, respectively. Generally, the cases appeared to increase during the study period, with a significant decline in 2018 (Fig. 1).

### Age assessment of all brain tumors

During the study period, 345 brain tumor records were retrieved. Age data were missing for 33 records; hence, 312 records were included for age analyses. The mean age for the pediatrics (≤ 20 years) and adults was 9 (± 5 SD) and 45 (± 14 SD) years, respectively. Eighty-eight (28.2%) and 224 (71.8%) tumors were diagnosed among pediatrics and adults, respectively. Most tumors, 60 (19.2%) were reported in patients aged ≤ 10 years, followed by 55 (17.6%), 48 (15.4%), and 47 (15.1%) in patients aged 31–40, 51–60, and 41–50, years, respectively. In both pediatrics and adults, most tumors were diagnosed in females aged ≤ 10 years and 31–40 years, respectively (Table 1).

**Table 1:** Distribution of brain tumors according to age and sex

Age category	Female n (%)	Malen (%)	Total n (%)
≤10 years	33 (19.3)	27 (19.1)	60 (19.2)
11–20 years	15 (8.8)	13 (9.2)	28 (9)
21–30 years	19 (11.1)	21 (14.9)	40 (12.8)
31–40 years	32 (18.7)	23 (16.3)	55 (17.6)
41–50 years	29 (17)	18 (12.8)	47 (15.1)
51–60 years	28 (16.4)	20 (14.2)	48 (15.4)
≤61 years	15 (8.8)	19 (13.5)	34 (10.9)
Total	171 (100)	141 (100)	312 (100)

**Fig. 2:**
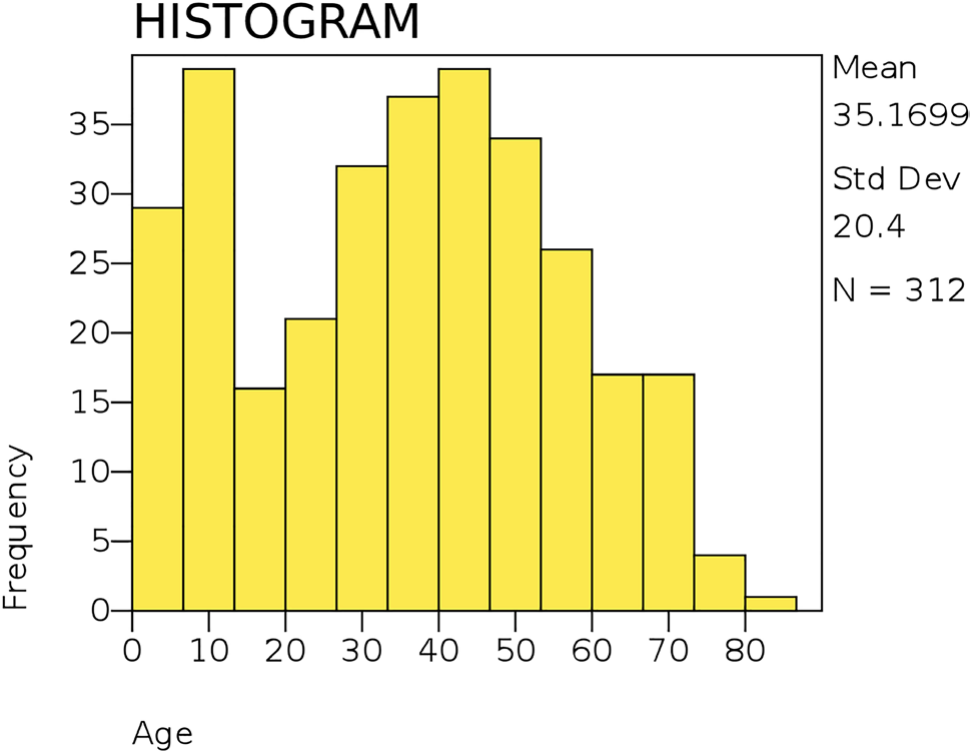
Histogram showing the distribution of brain tumors according to age

There was a bimodal distribution of the brain tumors with peaks at 5–15 years and 40–45 years (Fig. 2).

### Assessment of brain tumors according to sex

Of 345 cases, 190 (55.1%) were diagnosed in females, while 155 (44.9%) were diagnosed among males. Cumulatively, meningiomas were the most prevalent tumors followed by gliomas, medulloblastomas, brain metastasis and pituitary adenomas with 128 (37.1%) and 118 (34.%), 26 (7.5%), 19 (5.5), and 15 (4.3), respectively. Meningiomas were most common in females, with 86 (50.6%) cases, followed by gliomas, with 48 (28.2%) cases. In males, gliomas were most common, with 59 (40.4%) cases, followed by meningiomas, with 29 (19.9%) cases (Table 2).

**Table 2:** Histological distribution of brain tumors according to sex

Tumor type	Female n (%)	Male n (%)	Total N (%)
Meningioma	96 (50.5)	32 (20.6)	128 (37.1)
Glioma	55 (28.9)	63 (40.6)	118 (34.2)
Medulloblastoma	10 (5.3)	16 (10.3)	26 (7.5)
Brain metastasis	10 (5.3)	9 (5.8)	19 (5.5)
Pituitary adenoma	6 (3.2)	9 (5.8)	15 (4.3)
Ependymoma	4 (2.1)	6 (3.9)	10 (2.9)
Hemangioblastoma	1 (0.5)	9 (5.8)	10 (2.9)
Craniopharyngioma	3 (1.6)	5 (3.2)	8 (2.3)
PNET	1 (0.5)	2 (1.3)	3 (0.9)
Choroid plexus papilloma	2 (1.1)	0 (0)	2 (0.6)
Hemangiopericytoma	0 (0)	2 (1.3)	2 (0.6)
Lymphoma	1 (0.5)	1 (0.6)	2 (0.6)
Germinoma	0 (0)	1 (0.6)	1 (0.3)
Schwannoma	1 (0.5)	0 (0)	1 (0.3)
Total	190 (100)	155 (100)	345 (100)

*PNET: primitive neuroectodermal tumor*

### Assessment of pediatric brain tumors

Gliomas (43, 48.9%), followed by medulloblastomas (21, 23.9%), were the most common tumors of all pediatric tumors. Most, 50 (56.8%) pediatric tumors were benign (WHO grade 1 and 2), whereas 38 (43.2%) were malignant (WHO grade 3 and 4). Low-grade gliomas and ependymomas were the most common benign tumors, with 31 (62%) and 7 (14%) cases, respectively. Medulloblastomas and high-grade gliomas were the most common malignant tumors, with 21 (55.3%) and 10 (31.6%) cases, respectively (Table 3).

**Table 3:** Distribution of pediatric brain tumors according to the WHO Classification of CNS tumors

Diagnosis	WHO Grade 1 n (%)	WHO Grade 2 n (%)	WHO Grade 3 n (%)	WHO Grade 4 n (%)	Total N (%)
Glioma	28 (68.3)	3 (33.3)	3 (75)	9 (26.5)	43 (48.9)
Medulloblastoma	0 (0)	0 (0)	0 (0)	21 (61.8)	21 (23.9)
Ependymoma	2 (4.9)	5 (55.6)	1 (25)	0 (0)	8 (9.1)
Craniopharyngioma	4 (9.8)	0 (0)	0 (0)	0 (0)	4 (4.5)
Meningioma	2 (4.9)	1 (11.1)	0 (0)	0 (0)	3 (3.4)
PNET	0 (0)	0 (0)	0 (0)	3 (8.8)	3 (3.4)
Choroid plexus papilloma	2 (4.9)	0 (0)	0 (0)	0 (0)	2 (2.3)
Germinoma	0 (0)	0 (0)	0 (0)	1 (2.9)	1 (1.1)
Hemangioblastoma	1 (2.4)	0 (0)	0 (0)	0 (0)	1 (1.1)
Pituitary adenoma	1 (2.4)	0 (0)	0 (0)	0 (0)	1 (1.1)
Schwannoma	1 (2.4)	0 (0)	0 (0)	0 (0)	1 (1.1)
Total	41 (100)	9 (100)	4 (100)	34 (100)	88 (100)

*PNET: primitive neuroectodermal tumor*

### Distribution of adult brain tumors

Meningiomas, 107 (47.8%) were the most common tumors of all adult BTs, followed by gliomas, brain metastasis, and pituitary adenomas with 70 (31.3%), 17 (7.6%), and 11 (4.9%), respectively. Most 157 (70.1%) adult tumors were benign, whereas 67 (29.9%) were malignant. Meningiomas and low-grade gliomas were the most common benign tumors, with 106 (67.5%) and 26 (16.6%) cases, respectively. High-grade gliomas and brain metastases were the most common malignant tumors, with 44 (65.7%) and 17 (25.4%) cases, respectively (Table 4).

**Table 4:** Distribution of adult brain tumors according to the WHO Classification of CNS tumors

Diagnosis	WHO Grade 1 n (%)	WHO Grade 2 n (%)	WHO Grade 3 n (%)	WHO Grade 4 n (%)	Total N (%)
Meningioma	97 (75.8)	9 (31)	1 (33.3)	0 (0)	107 (47.8)
Glioma	7 (5.5)	19 (65.5)	1 (33.3)	43 (67.2)	70 (31.3)
Brain metastasis	0 (0)	0 (0)	0 (0)	17 (26.6)	17 (7.6)
Pituitary adenoma	11 (8.6)	0 (0)	0 (0)	0 (0)	11 (4.9)
Hemangioblastoma	8 (6.3)	0 (0)	0 (0)	0 (0)	8 (3.6)
Craniopharyngioma	4 (3.1)	0 (0)	0 (0)	0 (0)	4 (1.8)
Medulloblastoma	0 (0)	0 (0)	0 (0)	3 (4.7)	3 (1.3)
Ependymoma	0 (0)	1 (3.4)	1 (33.3)	0 (0)	2 (0.9)
Hemangiopericytoma	1 (0.8)	0 (0)	0 (0)	0 (0)	1 (0.4)
Lymphoma	0 (0)	0 (0)	0 (0)	1 (1.6)	1 (0.4)
Total	128 (100)	29 (100)	3 (100)	64 (100)	224 (100)

### Distribution of gliomas according to age and sex

Of all tumors assessed by age, 113 were gliomas; of these, 54 (47.8%) were diagnosed in female patients, whereas 59 (52.2%) were diagnosed in males. Among females, most cases, 15 (27.8%) cases were reported in patients aged ≤ 10 years, followed by those between 11 and 20 years, with 10 (8.8%) cases. Among males, most, 11 (18.6%) cases were reported in patients aged ≤ 10 years, followed by those between 31 and 40 and 51–60 years with 10 (16.9%) each (Table 5).

**Table 5:** Distribution of gliomas according to age and sex

Age category	Female n (%)	Male n (%)	Total n (%)
≤10 years	15 (27.8)	11 (18.6)	26 (23)
11–20 years	10 (8.8)	7 (11.9)	17 (15)
21–30 years	7 (13)	5 (8.5)	12 (10.6)
31–40 years	7 (13)	10 (16.9)	17 (15)
41–50 years	4 (7.4)	9 (15.3)	13 (11.5)
51–60 years	8 (14.8)	10 (16.9)	18 (15.9)
≤61 years	3 (5.6)	7 (11.9)	10 (8.8)
Total	54 (100)	59 (100)	113 (100)

### Distribution of meningiomas according to age and sex

Of all tumors assessed by age, 110 were meningiomas. 85 (77.3%) were diagnosed in female patients, whereas 25 (22.7%) were diagnosed in male patients. Among females, most, 25 (29.4%) cases were reported in patients aged between 31 and 40 years, followed by those between 41 and 50 years, with 21 (24.7%) cases. Among males, 7 (28%) cases were reported in patients aged 51–60 years, followed by those between 31 and 40 years with 6 (24%) cases (Table 6).

**Table 6:** Distribution of meningiomas according to age and sex

Age category	Female vn (%)	Male n (%)	Total n (%)
≤10 years	2 (2.4)	0 (0)	2 (1.8)
11–20 years	1 (1.2)	0 (0)	1 (0.9)
21–30 years	10 (11.8)	4 (16)	14 (12.7)
31–40 years	25 (29.4)	6 (24)	31 (28.2)
41–50 years	21 (24.7)	3 (12)	24 (21.8)
51–60 years	17 (20)	7 (28)	24 (21.8)
≤61 years	9 (10.6)	5 (20)	14 (12.7)
Total	85 (100)	25 (100)	110 (100)

## Discussion

In this study, brain tumor cases showed an increasing trend over the years. The highest number of cases was reported in 2019; whereas a decline was observed in 2018. The low cases prior to 2019 may be attributed to various challenges at the institutional and health system challenges, including inadequate ICU beds, fewer surgery days due to less theaters available for neurosurgery, prioritization of other surgical emergencies over elective neurosurgeries, and sporadic nationwide health service disruption due to industrial actions [24]. Subsequently, dedicated neurosurgical theaters were set up with daily neurosurgery electives, which may explain the higher cases in 2019. However, these cases may not reflect the true burden of BTs in Kenya considering KNH remains the main neurosurgical and neurooncological care center. Patient factors, such as healthcare seeking behaviors, health system challenges, including service delivery, health workforce, health system financing, and neurosurgical and neurooncological care are largely overlooked in resource-limited settings, including Kenya [25]. This may impair health care and service delivery for brain tumors. There is also an underinvestment in healthcare infrastructure and brain tumor research in developing countries compared to developed countries [26]. There is acute shortage of comprehensive neurooncological care, including radiologists, neurooncologists, and neurosurgeons with dismal ratio of 1 neurosurgeon for 2.4 million people in East Africa, which Kenya is part of [27]. All these challenges may explain the persistently low incidence rates reported in the African region [1, 28].

In this study, the mean age for the pediatrics (≤ 20 years) and adults was 9 (± 5 SD) and 45 (± 14 SD) years, respectively. Most tumors, 71.8% tumors were diagnosed among adults. There was a bimodal distribution of the BTs with peaks at 5–15 years and 40–45 years. Most tumors, 60 (19.2%) were reported in patients aged ≤ 10 years, followed by 55 (17.6%), 48 (15.4%), and 47 (15.1%) in patients aged 31–40, 51–60, and 41–50, years, respectively. In both pediatrics and adults, most tumors were diagnosed in females aged ≤ 10 years and 31–40 years, respectively. This study’s mean age for pediatric BTs was relatively same to the current SEER report (United States), which reported a mean age of 8 years [29]. However, the mean age varies significantly according to the specific childhood brain tumors (CBTs) [30]. This study’s mean for adult BTs is relatively similar to another study that was conducted at the same facility in 2014 that reported a mean of 40.63 ± 15.36 years [31]. Generally, the distribution of BTs according to age was relatively similar to the local study cited above [31] and with the CBTRUS findings [32]. In this study, two peaks were observed in both childhood and adulthood, like other studies [2, 6]. The bimodal incidence of BTs is attributed to the likelihood of certain BTs occurring at a certain age due to the associated biological, genetic, and environmental risk factors [2]. Childhood tumors, such as embryonal tumors and astrocytomas, are more common in children and may have genetic predispositions [6, 8, 30]. On the other hand, malignant gliomas, meningiomas, and pituitary adenomas are common in adults [6]. Adult BTs are also associated with the relatively long duration of exposure needed for neoplastic transformation [2, 8]. Biological factors, such as hormones are attributed to high adult glioma and meningioma cases [8]. In this study, BTs diagnosed in males were 46.2% versus 53.8% in females; thus, the overall male-to-female (M:F) ratio was 1:1.6. This compared unfavorably with other African studies that reported an M:F of 1:1 [33, 34]. This could be attributed to the relatively high frequency of meningiomas diagnosed in females in this study. Gliomas were most diagnosed in males, whereas meningiomas were the most diagnosed brain tumor type in females. Gliomas had M:F ratios of 1:1.1, whereas meningiomas had a M:F ratio of 1:3.4. These findings are consistent with local and international studies [31, 35].

Gliomas and medulloblastomas were the most common pediatric tumors in this study. In addition to gliomas and medulloblastomas, other studies have reported craniopharyngiomas and ependymomas among the most frequent pediatric tumors [6]. Most pediatric tumors in this study were benign, like other studies [36]. In this study, gliomas were the most common benign pediatric BTs, whereas medulloblastomas were the most malignant brain pediatric tumors. Low-grade gliomas are the most common childhood tumors, and rarely become malignant [37]. In this study, medulloblastomas were the second most frequent of all pediatric tumors, but the most common malignant childhood BTs and were most frequently diagnosed in males, like a current review on pediatric tumors studies [30]. Medulloblastomas are the commonest component of the heterogenous group of malignant tumors known as CNS embryonal tumors [38]. CNS embryonal tumors were previously categorized as primitive neuroectodermal tumors (PNETs); however, molecular characterization necessitated the reclassification [30]***.*** Ependymomas were the third most common tumors at 8% of all pediatric tumors in this study. This is similar to the generally reported frequency of approximately 5–10% [30]. Some of the risk factors associated with pediatric tumors include genetic factors, exposure to ionizing radiation (IR), non-chromosomal structural birth defects, high socioeconomic position, and high birth weight [8, 39]. Genetic predisposition is a known risk factor in the occurrence of pediatric ependymomas, medulloblastomas and gliomas [8, 30]. However, low-grade gliomas with isocitrate dehydrogenase 1 gene (IDH1) and IDH2 mutations, which transform to malignancy are rarer in pediatrics [40]. Longer telomere length and European ancestry are associated with increased risk for ependymomas [6]. However, genetic association studies in CBTs have not been conducted in other tumors; hence, the contribution of genetic factors is unknown in these tumors [6].

Moderate to high radiation doses are known causes of brain tumors, including CBTs [8, 39]. However, the risk of developing brain cancer from low-dose diagnostic or therapeutic radiation is debatable. The carcinogenic effects of IR are heightened in children, particularly in younger children [8]. Postnatal exposure to computed tomography was associated with increased risk for BTs [39]. Childhood exposure to therapeutic radiation for leukemia is also associated with development of BTs in adulthood [8]. Reverse causation may limit these findings as pediatrics with preexisting cancer or at a higher risk for cancer are likely to undergo computed tomography of the head [8]. During pregnancy, exposure of the mother to diagnostic radiation is also thought to increase brain cancer risk [8]. The tumors that may be induced by IR include gliomas and meningiomas [41]. Generally, non-chromosomal structural birth defects are known risk factor for CBTs with more preponderance in children aged ≤ 5 years with cancer [8]. CNS anomalies have a higher risk of CBTs. Birth weight > 4000 g is also associated with higher risk of pediatric embryonal tumors and astrocytomas [6]. However, other studies reported no associations between birth weight and CBTs [42]. There is limited evidence on the contribution of these risk factors to CBTs from low-resource settings, particularly Africa.

Overall, most adult BTs cases in this study were reported in patients aged 40–60 years. High cases were reported in female patients between 31 and 60 years in this study. Meningiomas and gliomas accounted for most of the adult BTs, with a cumulative prevalence of 79.1%. This is like other studies, both locally [31, 43] and globally [32, 33, 44]. Most adult tumors were benign, with meningiomas being the most frequent. Meningiomas mostly occur in patients in their fourth and fifth decades of life, with more female cases than males [45]. High-grade gliomas were the most frequent malignant adult tumors, followed by brain metastases. High-grade gliomas were most reported in males. In this study, metastatic tumors to the brain were the fourth most frequently reported of all tumors, with all cases in adults. This is consistent with other studies [31, 33, 46]. The increased prevalence of brain metastases is attributed to improved cancer care, and thus, cancer patients live longer, with an estimated 20–40% likelihood of developing BTs [14]. Increased access to imaging may also explain the increasing diagnosis of brain metastasis among cancer patients. The risk factors associated with adult BTs include genetic mutations, ancestry, increased leucocyte length, human leucocyte antigen (HLA) haplotypes, hormonal factors, environmental exposures, and higher socioeconomic status [6, 47]. On the contrary, some factors such as use of aspirin, statins, and history of respiratory allergies decrease the risk of adult BTs, particularly gliomas [8, 48]. Most risk association studies have been conducted in high-grade gliomas since they are the most frequent malignant BTs. Most tumors, particularly gliomas do not have a family history of glioma; however, approximately 5% are familial [8]. Studies on genetic polymorphisms in gliomas and meningiomas, pituitary adenomas, and primary CNS lymphoma have found various single nucleotide polymorphisms associated with these BTs, particularly in the European and East Asian populations [6, 49]. Increased leukocyte telomere length has been implicated in meningiomas and gliomas [50, 51]. Neurofibromatosis type 2 (NF2) mutation is also associated with meningiomas [45]. However, genetic studies are limited in Africa, including Kenya.

High-dose IR is one of the environmental exposure factors that has been widely evaluated in adult BTs and has been implicated in gliomas and meningiomas [6]. However, this may not account for the high burden of adult BTs. Studies on other environmental exposures, including radiofrequency fields emitted by cellular phones, extremely low frequency (ELF) magnetic fields, electromagnetic fields (EMF) from power lines, and other non-radiation exposures have reported conflicting results or no associations with adult BTs [52, 53]. The contribution of these environmental exposure in Kenya is unknown. Higher socioeconomic position (SEP) has been linked to higher risk for adult BTs, particularly gliomas [54, 55]. This may be due to diagnostic bias where underreporting may occur in patients with lower SEP. Another explanation is that immune exposures, particularly allergy and infections and health-seeking behavior may be influenced by SEP [6, 8]. This may explain the varied burden of BTs between higher SEP and lower SEP settings, such as Kenya. Sex hormones are hypothesized to play role in the gender-associated prevalence in meningiomas and gliomas. Estrogen and progesterone are attributed to the high frequency of meningiomas in women [47, 56]. On the other hand, testosterone is implicated in the high glioma prevalence in males [57]. Studies on the association of estrogen and progesterone and meningiomas in premenopausal and perimenopausal women have reported conflicting results [58]. A population-based case–control study reported a marginal association between meningioma and exogenous hormone exposure from oral contraceptives and hormone replacement therapy. However, other studies found no association between meningioma and hormonal exposures [59, 60]. Other studies have reported protective role of endogenous hormones against meningiomas in premenopausal women [61]. Glioblastomas, which comprise high-grade gliomas are associated with testosterone, whereas progesterone and estradiol are thought to protect against glioblastomas [57]. Most associations studies between risk factors and brain tumors have been conducted in developed countries. Further research is needed, more so in low-resource settings to establish the association of risk factors to the existing pediatric and adult brain tumors.

## Study limitations

This study was dependent on the completeness of the recorded data. There was a lack of data on some variables, including tumor location, ancestry/ethnicity, and race. In addition, the lack of immunohistochemical and molecular classification of brain tumors limited the comparison of these findings to international studies.

## Conclusion

This study highlights the existing burden of brain tumors in Kenya. Data from KNH may be representative of the national burden of brain tumors. This study lays a foundation for subsequent clinical and epidemiological studies and the need to adopt existing reporting standards to help realize a complete picture of the burden of brain tumors in Kenya.

## Recommendations

This study recommends adherence to reporting recommendations to diagnose brain tumors. Building capacity to enable performance of the recommended tests, including immunohistochemistry and molecular studies, will help improve reporting and allow comparison to global studies.

## Data Availability

All data generated or analyzed during this study are included in this published article.
